# Optic Disc Vascular Density in Normal-Tension Glaucoma Eyes with or without Branch Retinal Vessel Occlusion

**DOI:** 10.3390/jcm10122574

**Published:** 2021-06-10

**Authors:** Jiwon Baek, Soo-Ji Jeon, Jin-Ho Kim, Chan-Kee Park, Hae-Young L. Park

**Affiliations:** 1 Department of Ophthalmology, Bucheon St. Mary’s Hospital, College of Medicine, The Catholic University of Korea, Bucheon 14647, Korea; md.jiwon@gmail.com (J.B.); dmtko1@naver.com (J.-H.K.); 2 Department of Ophthalmology, Eunpyeong St. Mary’s Hospital, College of Medicine, The Catholic University of Korea, Seoul 03312, Korea; sj8801@gmail.com; 3 Department of Ophthalmology, Seoul St. Mary’s Hospital, College of Medicine, The Catholic University of Korea, Seoul 06591, Korea; ckpark@catholic.ac.kr

**Keywords:** OCTA, vessel density, disc, peripapillary, NTG, BRVO

## Abstract

We analyzed the vascular densities (VDs) of the optic disc areas in eyes with normal-tension glaucoma (NTG) according to their branch retinal vessel occlusion (BRVO) status. The VDs of the optic discs and peripapillary areas of 68 NTG patients with BRVO (BRVO group; BRVO eyes and fellow eyes) and 37 patients with NTG alone (control eyes) were measured on angiographic images obtained via swept-source optical coherence tomography angiography. VDs were compared among groups and correlations were assessed. The VD of the optic disc large vessel was the highest in BRVO eyes, followed by the fellow eyes and controls (all *P* < 0.05). Conversely, small and medium vessel VD was in the opposite order (all *P* < 0.05). Large vessel VD was negatively correlated with small and medium vessel VD (r = −0.697, *P* < 0.001). Peripapillary VD was lower in the BRVO eyes than in the control and fellow eyes (*P* < 0.001 and *P* = 0.861, respectively). In conclusion, significant changes in the distribution of VDs for optic disc larger vessel and small and medium vessels were observed in both eyes of NTG patients with BRVO, compared to NTG patients without BRVO.

## 1. Introduction

An association between retinal vein occlusion (RVO) and glaucoma has been documented in many previous studies [1,2,3,4,5,6,7,8]. The prevalence of normal-tension glaucoma (NTG) has been reported to be higher in eyes with RVO than in the general population [1]. This pathogenesis may be explained by abnormal disc anatomy, elevated intraocular pressure (IOP), and the effects of various vascular factors. In the Ocular Hypertension Treatment Study, a greater cup-to-disc ratio was associated with RVO development in patients with elevated IOP [9]. Changes in disc hemodynamics may also link RVO and glaucoma, especially in eyes with NTG; such changes play pathogenic roles in both diseases [10,11,12].

Recently, optical coherence tomography angiography (OCTA) has been extensively used to analyze the vascular structures of the retina and disc area; OCTA facilitates the detailed, noninvasive evaluation of retinal and choroidal microvascular structures. The results of OCTA studies have supported the notion that hemodynamic changes may trigger glaucoma. Such studies have revealed reduced vascular densities (VDs) in the optic disc and peripapillary area in eyes with glaucoma, including NTG [13,14,15,16,17]. The recent publication of a meta-analysis of OCTA VDs in glaucoma patients confirmed a significant reduction in the mean peripapillary, whole optic disc, and inside-disc VDs [18]. Reduced VD has been associated with disease severity and progression [19,20,21].

Given that pathogenic hemodynamic parameters are shared by eyes with NTG and eyes with RVO, an analysis of vessel structures around the disc in patients with these diseases might afford useful insights into the roles played by vascular changes. Here, we identified changes in the vascular structures of the optic disc and peripapillary area and we used OCTA to quantitatively analyze the VDs of eyes with NTG, according to their branch retinal vein occlusion (BRVO) status.

## 2. Materials and Methods

This observational case-control study was performed using medical records. The study was approved by the Institutional Review Board of Seoul St. Mary’s Hospital (KC18RESI0852). Patients were informed of the study, but the requirement for written informed consent was waived because of the retrospective nature of the study. The study was conducted in accordance with the tenets of the Declaration of Helsinki.

### 2.1. Patients

Consecutive patients with NTG (with or without BRVO) in both eyes who visited Seoul St. Mary’s Hospital (Seoul, Korea) between March 2018 and March 2020 were enrolled. The number of patients in each group was decided using a two-tailed trial for 80% power and 5% significance. NTG diagnostic criteria were: (1) glaucomatous optic disc change (i.e., rim thinning, disc hemorrhage, rim notch, or vertical cup-to-disc ratio greater than that of the other eye by ≥0.2-fold) and glaucomatous visual field (VF) loss (i.e., pattern standard deviation (*P* < 0.05) or glaucoma hemifield test result (*P* < 0.01) outside the normal limits, exhibiting a consistent pattern in the Bjerrum areas of both VFs); (2) maximum IOP < 22 mm Hg (without glaucoma medications) as determined by repeated measurements performed on different days; and (3) an open angle on gonioscopic examination.

BRVO was defined as retinal venous obstruction in a localized area of the retina, characterized by scattered superficial and deep retinal hemorrhages, venous dilation, intraretinal microvascular abnormalities, and occluded and sheathed retinal venules. BRVO was diagnosed at the time of initial NTG diagnosis or during NTG follow-up.

The exclusion criteria were: (1) spherical refraction > ±6.0 diopters; (2) glaucoma with BRVO in both eyes; (3) history of any retinal disease other than BRVO; (4) history of eye trauma or surgery, with the exception of uncomplicated cataract surgery; and (5) any optic nerve disease other than glaucoma.

All patients underwent a complete ophthalmic examination, including assessment of best-corrected visual acuity (BCVA) and refractive error; slit-lamp biomicroscopy; gonioscopy; IOP measurement using Goldmann applanation tonometry; axial length assessment via ocular biometry (IOLMaster; Carl Zeiss Meditec, Dublin, CA, USA); stereoscopic photography and red-free fundus photography (Canon, Tokyo, Japan); Humphrey VF testing using the Swedish Interactive Thresholding Algorithm Standard 24-2 test (Carl Zeiss Meditec, Dublin, CA, USA); optical coherence tomography (Carl Zeiss Meditec, Dublin, CA, USA) of the retinal nerve fiber layer (RNFL); and OCTA of the disc area (DRI OCT Triton, Topcon Corporation, Tokyo, Japan). OCTA data regarding eyes with BRVO were obtained during the inactive phase of BRVO (i.e., in the absence of edema and active hemorrhage). BCVA was converted into the logarithm of the minimal angle of resolution to allow statistical analysis.

### 2.2. Image Analysis 

**Figure 1:**
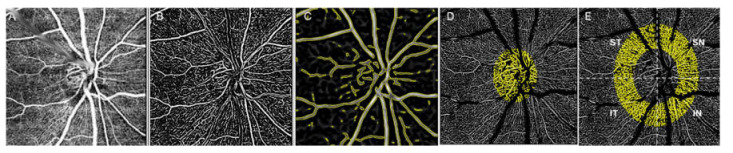
Measurement of vascular density: (**A**) En face, optical coherence tomography angiography image of 3 × 3-mm^2^ disc area showing vessels from two combined layers; (**B**) Vessels were traced using Frangi vesselness plug-in; (**C**) Large vessel areas were selected using Tubness plug-in. Regions of interest were selected in the optic disc (**D**) and peripapillary area (**E**). Vascular density was calculated by dividing vessel area by region of interest.

En face OCTA images (3 × 3 mm^2^ in area, centered at the optic disc) were obtained for each eye. For eyes with macular edema and hemorrhage, OCTAs were taken after subsidence of fluid and hemorrhage. All images were acquired after confirmation of the flow signal shown on the OCT B-scan. For VD analysis, a combined image was automatically generated by ImageNet software (ImageNet 6, ver. 1.19.11030, Topcon Corporation, Tokyo, Japan) and the superficial and choriocapillaris layers were fused (Figure 1A). VD was quantified as follows using FIJI software (an expanded version of ImageJ ver. 1.51a, available at http://fiji.sc/Fiji, accessed on 10 June 2021). First, all vessels were traced using the Frangi vesselness plugin (Figure 1B). Large vessels were defined as retinal arteries and veins coming from and into the optic disc, and vessel areas were traced using the Tubness plugin (Figure 1C). Then, the total, small and medium, and large vessel VDs were measured in regions of interest of the optic disc and peripapillary area; the final figures were calculated by dividing the vessel area by the total region of interest (Figure 1D,E). Peripapillary VDs were subdivided into four quadrants: superotemporal (ST), superonasal (SN), inferonasal (IN), and inferotemporal (IT) (Figure 1E). The means of two measurements of each VD were used. Cases with media opacities were excluded. Only clear images with signal strength index >40 that did not exhibit blurring or artifacts attributable to motion were analyzed. 

### 2.3. Statistical Analysis

Continuous variables were marked as mean ± standard deviation. Statistical analysis was performed using SPSS Statistics (version 23.0.1; IBM Corp., Armonk, NY, USA). Independent t-test was used for the comparison between unpaired eyes, while paired t-test was used for paired eyes after confirmation of a normal distribution using Kolmogorov–Smirnov test. The Mann–Whitney U test and Wilcoxon signed rank test were employed when a normal distribution could not be confirmed. Categorical variables were compared between groups using the chi-squared test. Standardized adjustment for residuals was used as the post hoc test after the chi-squared test. Effect of treatment modality on VD was assessed using multinomial logistic regression. Correlation analysis was performed with Spearman rank correlation for continuous variables and univariate logistic regression for binary variables. A *p*-value < 0.05 was considered statistically significant.

## 3. Results

### 3.1. Patient Demographics and Clinical Features

In total, 68 NTG patients with BRVO (BRVO group; 68 eyes with BRVO and 68 fellow eyes) and 37 patients with NTG alone (control group; 74 control eyes) were included in this study. Four patients (5.6%) with low image quality were excluded. The mean patient age was 66.97 years. There were no significant differences in age, sex, diabetes mellitus status, cardiovascular disease status, or cerebrovascular disease status between the BRVO group and the control group (all *P* > 0.05). Hypertension was more common in the BRVO group than in the control group (*P* < 0.001). All the NTG patients were on treatment eyedrops: 30 were on prostaglandin analogues, 10 were on beta-blockers, 2 were on alpha-agonist, and 26 were on combination treatment. Patient demographics are summarized in Table 1.

The mean BCVA values were lower in BRVO eyes than in fellow eyes and control eyes (both *P* < 0.05). The mean VF deviation was lower in BRVO eyes compared to control eyes (*P* = 0.006). No other ocular parameters differed significantly between BRVO eyes, fellow eyes, and control eyes (all *P* > 0.05). Ocular parameters are summarized in Table 2.

### 3.2. Comparison of VDs Among Groups 

**Figure 2:**
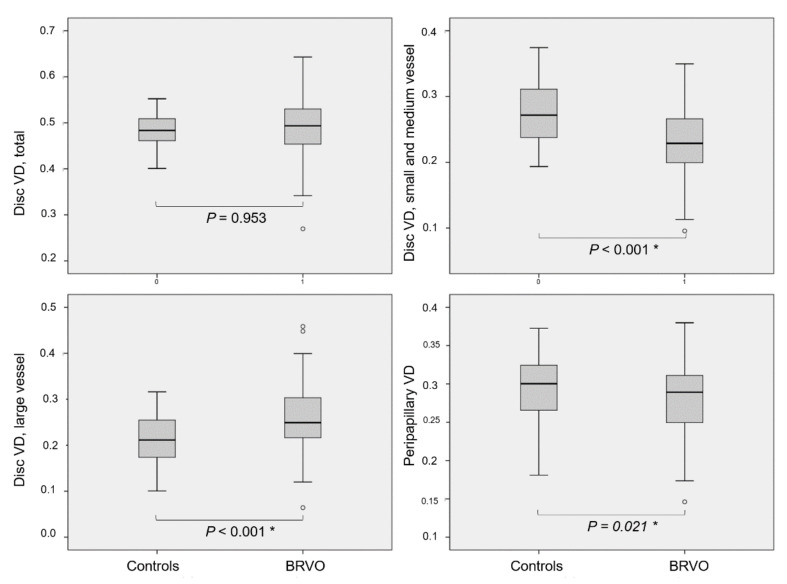
VDs of the disc and peripapillary areas of patients with NTG, according to BRVO status. Small and medium and large vessel VDs in the disc area differed significantly between patients with NTG, according to BRVO status (both *P* < 0.001). Peripapillary VD was lower in BRVO group than in control group (*P* = 0.021).* Statistically significant *P*-value with Mann–Whitney U test.

Although the total VD of the optic disc area did not differ between the BRVO group and the control group (0.485 ± 0.033 vs. 0.484 ± 0.082; *P* = 0.835), the small and medium vessel and large vessel VDs in the disc area significantly differed between the groups (0.232 ± 0.056 vs. 0.274 ± 0.045 and 0.260 ± 0.068 vs. 0.210 ± 0.053; both *P* < 0.05). Peripapillary VD also significantly differed between the groups (0.282 ± 0.046 vs. 0.296 ± 0.038; *P* = 0.021) (Figure 2). Treatment modality did not affect the VDs of the disc or peripapillary area (both *P* = 0.999).

When eyes were divided into control eyes, BRVO eyes, and fellow eyes, the large vessel VD was highest in BRVO eyes, followed by the fellow eyes and control eyes; the small and medium vessel VD exhibited the opposite order (all *P* < 0.05). The BRVO eyes exhibited lower small and medium vessel and peripapillary VDs, and higher large vessel VDs, compared to both the control eyes and fellow eyes (all *P* < 0.05). The fellow eyes exhibited a higher large vessel VD and a lower small and medium vessel VD than did the control eyes (*P* < 0.001 and = 0.020, respectively) (Table 3).

### 3.3. Correlation Analysis of VDs

**Figure 3:**
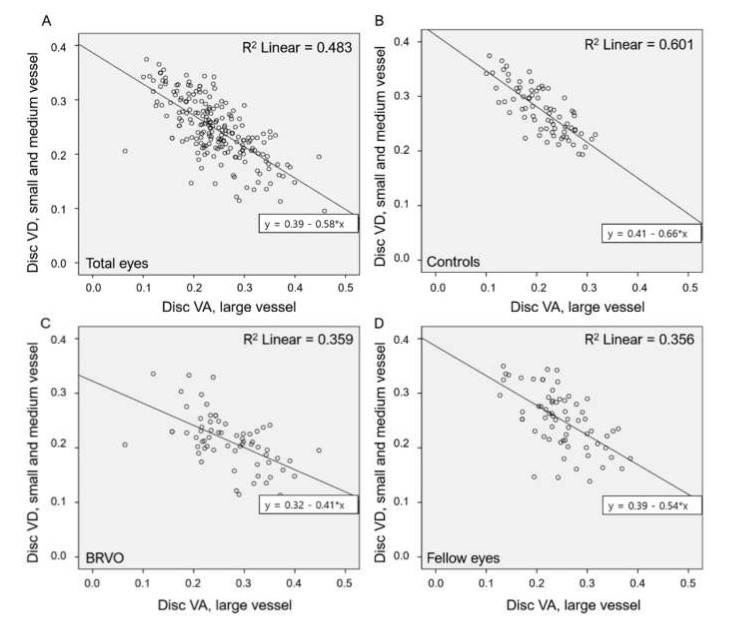
Correlations between large and small and medium vessel VDs in the optic disc area: (**A**) Large vessel VD correlated negatively with small and medium vessel VD; (**B**–**D**) This correlation remained consistent when patients were divided into groups.

**Figure 4:**
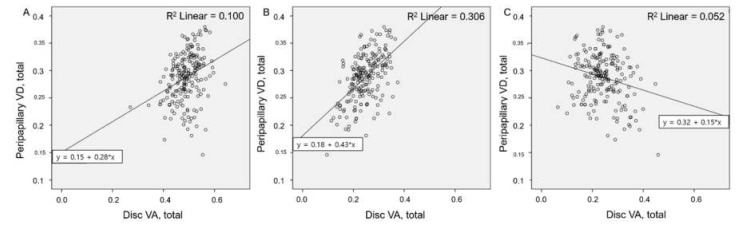
Correlation between disc and peripapillary VDs: (**A**,**B**) Total and small and medium vessel VDs of the optic disc area were correlated positively with peripapillary VD; (**C**) Larger vessel VD of the optic disc area was correlated negatively with peripapillary VD.

Total, small and medium vessel, and peripapillary VDs were negatively correlated with BCVA values (r = −0.147, −0.237, and −0.311; *P* = 0.034, 0.001, and <0.001, respectively). Large vessel VD was positively correlated with BCVA (r = 0.159, *P* = 0.023). Disc VDs were correlated with refractive error and axial length (all *P* < 0.05) (Table 4). Large vessel VD was negatively correlated with small and medium vessel VD (r = −0.697, *P* < 0.001) (Figure 3A). This correlation persisted when eyes were divided into groups (r = −0.775, −0.559, and −0.603; all *P* < 0.05) (Figure 3B–D). Peripapillary small and medium vessel VDs were positively correlated with the total and small and medium vessel VDs of the optic disc (r = 0.307 and 0.545; both *P* < 0.05) and negatively correlated with the large vessel VD of the optic disc (r = −0.226; *P* = 0.001) (Figure 4).

## 4. Discussion

An understanding of hemodynamic changes in the optic disc area may yield valuable insights into the pathogenesis of NTG and BRVO. OCTA enables the noninvasive visualization of vessels in the retina and optic disc as well as the quantification of vessel parameters, thus imparting detailed information regarding hemodynamic changes associated with disease. Here, we quantitatively analyzed the VDs of optic disc vessels evident in OCTA images of eyes with NTG, according to their BRVO status; we compared these findings between and among groups. We found differences in the VDs of the optic disc and peripapillary area between eyes with NTG, according to their BRVO status; we also found correlations between the VDs of the large and small and medium vessels in the disc and peripapillary area. Our principal findings were that BRVO was associated with enhanced large vessel VD and reduced small and medium vessel VD in the optic disc. This was evident in both eyes with BRVO and fellow eyes. Furthermore, the large vessel and small and medium vessel VDs differed significantly between fellow eyes with NTG alone and control eyes, suggesting that such changes may contribute to NTG development in patients with BRVO. Peripapillary VD was significantly reduced only in eyes with NTG + BRVO, suggesting that the change may be attributable to BRVO.

The contributions of large and small and medium vessels to the disc VD differed between eyes with NTG, according to their BRVO status. In NTG patients with BRVO, the mean VD of large vessels was higher, whereas the mean VD of small and medium vessels was lower. As factors that might affect disc vasculature (e.g., age, comorbid diabetes, refractive error, and axial length; Table 4) were comparable among the groups, our interpretation may be valid. Considering the negative correlation between large and small and medium vessel densities observed in the current study, the engorgement of large vessels (especially veins) may be followed by the attenuation of small and medium vessels in eyes with BRVO. The peripapillary VDs were also lower in eyes with BRVO. The optic disc head occupies a limited space; thus, the enhanced large vessel volume caused by congestion may trigger the mechanical compression of small and medium vessels. However, the details of this underlying mechanism requires further investigation.

The fellow eyes in patients with BRVO exhibited a greater large vessel VD and a lower small and medium vessel VD compared to the control eyes, suggesting that underlying systemic factors affect both eyes in patients with BRVO. Hypertension is a possible contributing factor: this demographic differed between patients with BRVO and the control group. In a previous study, we showed that patients with BRVO exhibited more rapid glaucoma progression in their fellow eyes compared to patients with glaucoma who did not develop BRVO [22]. The difference in fellow eye hemodynamics between patients with BRVO and the control group, observed in the present study, might be relevant in this context.

Here, we also found that the fellow eyes of patients with BRVO exhibited a significantly lower small and medium vessel VD in the optic disc, compared with the control eyes. The peripapillary VD parameters did not differ between the fellow eyes in the patients with BRVO and the control eyes, suggesting that a small and medium vessel VD change in the optic disc may accelerate glaucoma progression in the glaucomatous fellow eyes of patients with BRVO [19]. Systemic factors may trigger changes in the disc VDs of both eyes, thereby increasing the proportion of large vessels in patients who develop BRVO, followed by more prominent changes. As this was a cross-sectional study, we could not determine whether the phenomenon was a cause or a result of BRVO.

Blood flows from two principal sources to the optic disc. The superficial layers of the optic nerve head (i.e., the RNFL) are supplied by the central retinal artery; the deeper layers (i.e., the prelaminar, lamina cribrosa, and retrolaminar regions) are supplied by the posterior ciliary artery [23]. Analysis of these respective layers would yield detailed information regarding whether the observed changes reflect alterations in the branches of the central retinal or posterior ciliary arteries. However, the resolution of the current OCTA systems is inadequate for such analysis; it may be achieved in the future by using more powerful angiographic imaging systems.

The peripapillary VDs of the entire region and all four quadrants were lower in eyes with BRVO than in fellow eyes and controls, consistent with the results of a previous study by Shin et al. [24]. Notably, Shin et al. reported that various peripapillary microvascular parameters were lower in the fellow eyes of patients with RVO. Most of the vessels visible on OCTA scans of the peripapillary area are retinal radial peripapillary capillaries [25]. These radial peripapillary capillaries branch from the central retinal artery; thus, a lower peripapillary VD can be largely explained by a reduction of perfusion from the central retinal artery, perhaps attributable to the venous engorgement of eyes with BRVO and the negative correlation between the large vessel VD of the optic disc and the peripapillary VD, despite the weak correlation. However, this may be less important than in the optic disc area, as the peripapillary area is not a limited space. Other possible causes of reduced central retinal artery perfusion include capillary attenuation attributable to vasospasm, atherosclerosis, or shunting.

Our study had the limitations inherent to all cross-sectional retrospective analyses. Systemic factors which are suspected to alter VD, such as blood pressure, were not available due to the retrospective nature of the study. As mentioned above, we could not investigate causal relationships between vessel changes and disease development; we showed only that hemodynamic changes are evident. However, to the best of our knowledge, this study was the first to quantitatively analyze disc vessel density in eyes with NTG, according to their BRVO status. As hemodynamic factors play important pathophysiological roles in both NTG and BRVO, we believe that these results deepen our understanding of the pathogenesis of both diseases. Additionally, although there may be a small chance of inaccurate vessel separation due to the inherent errors of the OCTA system, each step in the VD measurement method used in this study was automatized, therefore yielding high repeatability and reproducibility.

In conclusion, we measured the VDs of the optic disc area of eyes with NTG, according to their BRVO status; in eyes with BRVO, we revealed the enhancement of large vessel VD and reduction of small and medium vessel VD as well as the reduction of peripapillary VD. The large vessel VD was significantly enhanced and the small and medium vessel VD was significantly reduced in fellow eyes (with NTG alone) in patients with BRVO, suggesting that hemodynamic changes may be involved in the progression of NTG or BRVO in these patients. Our results suggest that the hemodynamics around the disc area differ in eyes with NTG, according to their BRVO status, and may be associated with disease development or progression. Prospective follow-up studies with larger samples are required to confirm our findings.

## Figures and Tables

**Table 1:** Demographic and clinical parameters of normal-tension glaucoma patients with BRVO and controls.

Variable	Control Group (*n* = 37)	BRVO Group (*n* = 68)	*p*-Value
Age (years), mean ± SD	66.14 ± 10.89	67.81 ± 10.63	0.285 ^a^
Sex (male/female), *n*	16/21	22/46	0.118 ^b^
Diabetes mellitus, *n* (%)	5 (14)	15 (22)	0.133 ^b^
Hypertension, *n* (%)	4 (11)	31 (46)	<0.001 ^b^ *
CVD (0: no/1: yes)	4 (11)	3 (4)	0.076 ^b^

BRVO: branch retinal vein occlusion; SD: standard deviation; CVD: cardio- or cerebrovascular disease. ^a^ Independent *t*-test between groups. ^b^ Chi-square test between groups. * *p*-value is significant.

**Table 2:** Ocular parameters of the groups.

	Control Eyes (*n* = 74)	BRVO Eyes (*n* = 68)	Fellow Eyes (*n* = 68)	*p*-Value ^a^	*p*-Value ^b^	*p*-Value ^c^
BCVA (logMAR), mean ± SD	0.08 ± 0.11	0.21 ± 0.26	0.11 ± 0.18	<0.001 *	0.114	<0.001 *
Refractive error (diopter), mean ± SD	−0.3 ± 2.66	−1.03 ± 2.34	−0.95 ± 2.45	0.071	0.144	0.598
Axial length (mm), mean ± SD	24.3 ± 1.28	24.02 ± 1.43	23.84 ± 1.27	0.0.64	0.052	0.761
IOP (mmHg), mean ± SD	14.34 ± 3.04	14.76 ± 3.4	14.84 ± 3.59	0.448	0.271	0.961
RNFL thickness (µm), mean ± SD	83.01 ± 17.27	80.07 ± 21.51	83.09 ± 20.38	0.863	0.902	0.853
VF mean deviation (dB), mean ± SD	−4.07 ± 5.79	−6.73 ± 7.56	−5.07 ± 6.92	0.006 *	0.075	0.665
Disc hemorrhage, *n* (%)	7 (9)	2 (3)	5 (7)	0.232	0.653	0.564

NTG: normal tension glaucoma; BRVO: branch retinal vein occlusion; BCVA: best-corrected visual acuity; SD: standard deviation; IOP: intraocular pressure; RNFL: retinal nerve fiber layer; VF: visual field. ^a^ Mann–Whitney U test comparing control eyes and BRVO eyes. ^b^ Mann–Whitney U test comparing control eyes and fellow eyes. ^c^ Wilcoxon signed rank test comparing BRVO eyes and fellow eyes. * *p*-value is significant.

**Table 3:** Vessel densities of the groups.

Vascular Density	Control Eyes (*n* = 74)	BRVO Eyes (*n* = 68)	Fellow Eyes (*n* = 68)	*p*-Value ^a^	*p*-Value ^b^	*p*-Value ^c^
Disc total VD	0.485 ± 0.033	0.469 ± 0.101	0.499 ± 0.051	0.835	0.031 *	0.007 *
Disc small and medium vessel VD	0.274 ± 0.045	0.212 ± 0.051	0.252 ± 0.054	<0.001 *	0.020 *	<0.001 *
Disc large vessel VD	0.210 ± 0.053	0.272 ± 0.075	0.248 ± 0.059	<0.001 *	<0.001 *	0.003 *
Peripapillary VD	0.296 ± 0.038	0.269 ± 0.045	0.296 ± 0.042	<0.001 *	0.861	0.017 *
Peripapillary VD ST	0.318 ± 0.057	0.277 ± 0.056	0.308 ± 0.051	<0.001 *	0.297	0.019 *
Peripapillary VD SN	0.273 ± 0.053	0.255 ± 0.055	0.286 ± 0.048	0.032 *	0.396	0.023 *
Peripapillary VD IN	0.288 ± 0.034	0.261 ± 0.053	0.278 ± 0.05	0.001 *	0.285	0.387
Peripapillary VD IT	0.316 ± 0.041	0.282 ± 0.054	0.308 ± 0.058	<0.001 *	0.452	0.017 *

BRVO: branch retinal vessel occlusion; VD: vascular density; ST: superotemporal; SN: superonasal; IN: inferonasal; IT: inferotemporal. ^a^ Mann–Whitney U test comparing control eyes and BRVO eyes. ^b^ Mann–Whitney U test comparing control eyes and fellow eyes. ^c^ Wilcoxon signed rank test comparing BRVO eyes and fellow eyes. * *p*-value is significant.

**Table 4:** Correlation between vascular density and clinical parameters.

Vascular Desity		Age ^a^	Sex ^b^	DM ^b^	HBP ^b^	CVD ^b^	BCVA ^a^
Disc total VD	Coefficient	−0.076	−0.076	−0.038	0.090	0.043	−0.147 *
	*p*-value	0.274	0.274	0.582	0.196	0.533	0.034
Disc small and medium VD	Coefficient	−0.305 *	−0.058	−0.204 *	−0.111	−0.019	−0.237 *
	*p*-value	0.000	0.409	0.003	0.111	0.790	0.001
Disc Large VD	Coefficient	0.163 *	0.023	0.096	0.135	0.041	0.159 *
	*p*-value	0.019	0.738	0.171	0.054	0.558	0.023
Peripapillary VD	Coefficient	−0.206 *	0.125	−0.112	−0.018	0.022	−0.311 *
	*p*-value	0.003	0.073	0.110	0.799	0.757	0.000
Peripapillary VD ST	Coefficient	−0.198 *	0.102	−0.161 *	−0.070	−0.042	−0.270 *
	*p*-value	0.004	0.146	0.021	0.320	0.552	0.000
Peripapillary VD SN	Coefficient	−0.166 *	0.038	−0.010	0.134	0.001	−0.319 *
	*p*-value	0.017	0.585	0.884	0.055	0.989	0.000
Peripapillary VD IN	Coefficient	−0.146 *	0.081	−0.087	−0.053	0.069	−0.244 *
	*p*-value	0.037	0.247	0.212	0.453	0.326	0.000
Peripapillary VD IT	Coefficient	−0.145 *	0.139 *	−0.128	−0.128	0.043	−0.204 *
	*p*-value	0.038	0.047	0.066	0.068	0.540	0.003
**Vascular Desity**		**Refractive Error ^a^**	**Axial Length ^a^**	**IOP ^a^**	**RNFL Thickness ^a^**	**VF Mean Deviation ^a^**	**Disc Hemorrhage ^b^**
Disc total VD	Coefficient	0.176 *	−0.211 *	−0.088	0.105	0.183 *	0.016
	*p*-value	0.011	0.002	0.207	0.132	0.008	0.821
Disc small and medium VD	Coefficient	−0.218 *	0.169 *	−0.047	−0.005	0.109	0.069
	*p*-value	0.002	0.015	0.505	0.940	0.118	0.326
Disc Large VD	Coefficient	0.236 *	−0.242 *	0.031	0.025	−0.028	−0.060
	*p*-value	0.001	0.000	0.654	0.716	0.695	0.393
Peripapillary VD	Coefficient	0.034	−0.065	−0.039	0.247 *	0.277 *	0.027
	*p*-value	0.630	0.356	0.581	0.000	0.000	0.697
Peripapillary VD ST	Coefficient	−0.015	−0.030	−0.044	0.114	0.234 *	0.116
	*p*-value	0.832	0.668	0.531	0.104	0.001	0.096
Peripapillary VD SN	Coefficient	0.032	−0.037	−0.007	0.254 *	0.162 *	0.057
	*p*-value	0.646	0.596	0.922	0.000	0.020	0.414
Peripapillary VD IN	Coefficient	0.054	−0.049	−0.029	0.176 *	0.233 *	−0.016
	*p*-value	0.437	0.486	0.682	0.011	0.001	0.825
Peripapillary VD IT	Coefficient	0.086	−0.092	−0.047	0.208 *	0.256 *	−0.076
	*p*-value	0.221	0.189	0.505	0.003	0.000	0.275

DM: diabetes mellitus; HTN: hypertension; CVD: cardio- or cerebrovascular disease; BCVA: best-corrected visual acuity; IOP: intraocular pressure; RNFL: retinal nerve fiber layer; VF: visual field; VD: vascular density; ST: superotemporal; SN: superonasal; IN: inferonasal; IT: inferotemporal. ^a^ Spearman rank correlation. ^b^ Logistic regression. * *P* < 0.01 (two-tailed).

## Data Availability

The datasets generated and/or analyzed during the current study are available from the corresponding author upon reasonable request.

## Abbreviations

VD	vascular density
NTG	normal-tension glaucoma
RVO	retinal vein occlusion
BRVO	branch retinal vein occlusion
IOP	intraocular pressure
OCTA	optical coherence tomography angiography
VF	visual field
ST	superotemporal
SN	superonasal
IN	inferonasal
IT	inferotemporal
